# Hospitalization Risks for Neurological Disorders in Primary Sjögren’s Syndrome Patients

**DOI:** 10.3390/jcm11071979

**Published:** 2022-04-01

**Authors:** Radjiv Goulabchand, Audrey Gabelle, Xavier Ayrignac, Nicolas Malafaye, Pierre Labauge, Danièle Noël, Jacques Morel, Camille Roubille, Lucie Barateau, Philippe Guilpain, Thibault Mura

**Affiliations:** 1 Internal Medicine Department, CHU Nîmes, University Montpellier, 30029 Nîmes, France; 2 School of Medicine, University Montpellier, 30060 Montpellier, France; a-gabelle@chu-montpellier.fr (A.G.); x-ayrignac@chu-montpellier.fr (X.A.); p-labauge@chu-montpellier.fr (P.L.); j-morel@chu-montpellier.fr (J.M.); c-roubille@chu-montpellier.fr (C.R.); l-barateau@chu-montpellier.fr (L.B.); p-guilpain@chu-montpellier.fr (P.G.); thibault.mura@chu-nimes.fr (T.M.); 3 INSERM U1183, Institute for Regenerative Medicine and Biotherapy (IRMB), University Montpellier, 34090 Montpellier, France; daniele.noel@inserm.fr; 4 Memory Research and Resources Alzheimer Center, Department of Neurology, Montpellier University Hospital, 34090 Montpellier, France; 5 INSERM U1061, Neuropsychiatry: Epidemiological and Clinical Research, University Montpellier, 34090 Montpellier, France; 6 CRC SEP, Department of Neurology, Montpellier University Hospital, 34090 Montpellier, France; 7 INSERM, Institute for Neurosciences of Montpellier (INM), University Montpellier, 34090 Montpellier, France; 8 Department of Medical Information, Montpellier University Hospital, 34295 Montpellier, France; n-malafaye@chu-montpellier.fr; 9 Department of Rheumatology, Montpellier University Hospital, 34295 Montpellier, France; 10 PhyMedExp, University Montpellier, INSERM U1046, CNRS UMR 9214, 34295 Montpellier, France; 11 Department of Internal Medicine, Montpellier University Hospital, 34295 Montpellier, France; 12 Sleep-Wake Disorders Unit, Department of Neurology, Montpellier University Hospital, 34090 Montpellier, France; 13 Department of Internal Medicine and Multi-Organic Diseases, Montpellier University Hospital, 34090 Montpellier, France; 14 Department of Biostatistics, Clinical Epidemiology, Public Health, and Innovation in Methodology, CHU Nîmes, University Montpellier, 30029 Nîmes, France

**Keywords:** primary Sjögren’s syndrome, dementia, multiple sclerosis, encephalitis, peripheral neuropathy

## Abstract

Primary Sjögren’s syndrome (pSS) can be associated with neurological and cognitive involvement, negatively affecting patients’ quality of life. The aim of this study was to assess whether pSS patients are at higher risk of hospitalization for neurological diseases. Through a nationwide retrospective study using the French Health insurance database (based on International Classification for Disease codes, ICD-10), we selected patients hospitalized with new-onset pSS between 2011 and 2018. We compared the incidence of hospitalization for dementia, multiple sclerosis (MS), encephalitis, and peripheral neuropathy with an age- and sex-matched (1:10) hospitalized control group. Adjusted Hazard Ratios (aHR) considered confounding factors, particularly socio-economic status and cardiovascular diseases. We analyzed 25,661 patients hospitalized for pSS, compared with 252,543 matched patients. The incidence of hospitalization for dementia was significantly higher in pSS patients (aHR = 1.27 (1.04–1.55); *p* = 0.018), as well as the incidence of hospitalization for MS, encephalitis, and inflammatory polyneuropathies (aHR = 3.66 (2.35–5.68), *p* < 0.001; aHR = 2.66 (1.22–5.80), *p* = 0.014; and aHR = 23.2 (12.2–44.5), *p* < 0.001, respectively). According to ICD-10 codes, pSS patients exhibited a higher incidence of hospitalization for dementia, encephalitis, MS, and peripheral neuropathies than controls. Physicians must be aware of these neurological risks to choose the most appropriate diagnostic work-up.

## 1. Introduction

Primary Sjögren’s syndrome (pSS) is a systemic autoimmune disease characterized by sicca syndrome and is responsible for ocular and mouth dryness and extra-glandular B lymphocytic infiltration. Besides the classical triad involving sicca syndrome, fatigue, and pain, pSS patients often exhibit neurological and cognitive troubles [1,2], with a prevalence varying from 10 to 60% [3,4,5]. The burden of these symptoms is high in pSS patients, with profound consequences on quality of life [6,7]. Moreover, these symptoms and conditions increase the healthcare consumption of these patients, especially among young patients [1]. Cognitive dysfunctions have been reported in a small series of European patients [5,8] and a large series of Taiwanese patients [9,10]. These two populations have different genetic, environmental, and cultural backgrounds, potentially altering the clinical presentation and epidemiology of cognitive complaints. Thus, there is a lack of epidemiological data concerning the association between pSS patients and dementia within the European population. A better understanding of the interplay between pSS, dementia, and other associated neurological disorders might help to tailor treatment strategies, notably through a multidisciplinary approach. Therefore, this study aimed to describe the incidence of hospitalization for dementia and other neurological disorders in a real-life population of French hospitalized pSS patients, in comparison to age- and sex- matched controls.

## 2. Materials and Methods

### 2.1. National Database Characteristics

We performed a historical paired exposed/unexposed cohort study by analyzing data from the French National Hospital discharge database (“programme de médicalisation des systèmes d’information”, PMSI). This database covers over 99% of the population (65 million people), collecting all hospital stays and their causes in private and public hospitals. The available data are: age, sex, entry and discharge dates, diagnosis codes associated with the hospitalization (according to the International Classification of Diseases (ICD-10), as registered by the treating physician), inpatient deaths, and insurance scheme (general or associated with patients with a low socioeconomic status (LSS)). These data are anonymous and linkable from one year to another for a given patient; thus, we were able to analyze data from a 10-year period from 1 January 2009 to 31 December 2018. Information on clinical, biological, pathological data, procedures, or treatments were not available in the database, including disease activity scores such as the EULAR Sjögren’s Syndrome Disease Activity Index (ESSDAI).

### 2.2. Primary Sjögren’s Syndrome Patients (pSS)

We included all hospitalized pSS patients with at least one ICD-10 code of SS (M350) over the study period; we excluded potential secondary SS (sSS, the complete list of corresponding ICD-10 codes is available in Supplementary Table S1). Patients with a first code of SS in 2009 or 2010 were excluded in order to have a minimum of two years of records prior to the index date, to identify the medical history of hospitalizations.

### 2.3. Control Group of Non-pSS Hospitalized Patients

From the same database, we randomly selected 10 hospitalized patients for each pSS, matched on age, sex, and entry date of hospitalization (+/−31 days). The random selection was performed through the SAS (Statistical Analysis Systems Enterprise Guide 7.1, SAS Institute, Cary, NC, USA) program and excluded pSS patients. To avoid selecting patients with acute critical and severe illnesses at the index date, we excluded patients in both groups (SS and control) who died within 90 days of the index date and their corresponding match(es). Excluding these patients meant that the control group presented a disease severity level more comparable to the pSS patients. This cohort has been described in previous studies [11,12].

### 2.4. Outcomes

We collected the first hospitalization stay for dementia, multiple sclerosis (MS), encephalitis, and Parkinson’s disease (PD), according to ICD-10 codes, that occurred after 90 days from the index date. We also searched for the first hospitalization for peripheral neuropathies (inflammatory polyneuropathies, other polyneuropathies, polyneuropathies in other classified diseases, and trigeminal neuralgia) and migraine. For each disease, subjects with a previous mention of the condition (according to the same ICD-10 codes) in hospitalizations prior to this date, and their matched patients, were excluded (incidence study). The complete list of ICD-10 codes used in the study is available in Supplementary Table S2).

### 2.5. Adjustment Factors

We searched for the past medical history of each patient, screening diagnostic codes associated with hospitalizations prior to the index date + 90 days for: (i) hypertension (combining primary and secondary high blood pressure); (ii) diabetes (combining type 1 and type 2 diabetes, malnutrition diabetes, and unspecified diabetes); (iii) obesity; (iv) cardiovascular diseases (CVD) (combining ischemic heart disease, stroke, aortic dissection, aortic and peripheral artery disease, and hypertensive chronic kidney disease); (v) PD; (vi) encephalitis; and (vii) MS (according to ICD-10 codes, Supplementary Table S2). For the dementia incidence analysis, adjustments were also performed for past psychiatric disorders (depression and anxiety). We also collected the prior annual rate of hospitalization and LSS (the presence of one of the insurance schemes specific to low income patients) [13,14].

### 2.6. Statistical Analysis

The incidence rates of neurological disorders were calculated in each exposure group and presented with 95% confidence intervals. A survival analysis by Cox proportional hazard models, with stratification on the matched subjects, was performed to compare the incidence of each studied condition between exposure groups. Follow-up time was calculated as the interval between the index date and the date of the first hospitalization for the reported condition, or until December 2018, whichever occurred first. To take into account competitive risks, the follow-up of patients who died before December 2018 without a hospitalization for neurological disorders was censored at the date of death [15]. We first built a crude model, then an adjusted model, taking into account potential confounding factors: LSS, medical history of hospitalization for hypertension, diabetes, obesity, and cardiovascular disease (Supplementary Table S2). For the analysis of dementia, we excluded patients with a history of PD. To evaluate whether the association between pSS and dementia was mediated by the onset of MS or encephalitis (that may also lead to cognitive deficiency), we performed additional models, adjusting for these parameters as time-dependent covariates. Statistical analyses were performed at the two-tailed α level of 0.05 using the Statistical Analysis Systems Enterprise Guide 7.1 (SAS Institute, Cary, NC, USA).

## 3. Results

### 3.1. Population

Our search identified 25,661 hospitalized patients with suspected pSS and 252,543 matched hospitalized control patients (87.7% female), with a mean age of 60.0 (±16.3) years. The main characteristics of the population are shown in Table 1. The median follow-up time was 3.96 years. The annual rate of hospitalization before the index date was higher in the pSS group than in the controls (*p* < 0.001). In the pSS group, 4.77% of patients were classed as LSS versus 3.31% in the control group (*p* < 0.001). The incidence of death was higher in pSS patients (Table 1). Past hospitalizations for PD were reported in 0.22% of pSS patients and 0.06% of matched controls (*p* < 0.001). Past hospitalizations for MS and encephalitis were reported in 0.76% and 0.42% in pSS patients versus 0.10% and 0.02% in matched controls (*p* < 0.001), respectively.

### 3.2. Incidental Hospitalization Risk for Dementia

The risk of hospitalization for dementia (excluding PD) was increased in pSS patients (aHR = 1.37; 95% CI: (1.18–1.58); *p* < 0.001). This increased risk was maintained after adjustment (aHR = 1.27 (1.04–1.55); *p* = 0.018) (Table 2), and also after including past psychiatric disorders (aHR = 1.25 (1.02–1.53); *p* = 0.030). Supplemental mediation analysis with adjustment for MS and encephalitis as time-dependent covariates did not change the strength of the association (respectively, aHR = 1.27 (1.04–1.56) and aHR = 1.27 (1.04–1.55)). The majority of patients with a new hospitalization for dementia were women (84%), with a mean age of 78.6 ± 7.4 years (median 79 (74–84)).

### 3.3. Incidental Hospitalization Risk for Associated Neurological Disorders

There was an increased risk of hospitalization for MS in the pSS group compared to control patients (aHR = 3.66 (2.35–5.68); *p* < 0.001), and for encephalitis (aHR = 2.66 (1.22–5.80); *p* = 0.014) (Table 2). In addition, the risks of hospitalization for inflammatory polyneuropathies, other polyneuropathies, or polyneuropathies classified in other diseases was increased in pSS patients (respectively aHR = 23.2 (12.2–44.5)), aHR = 15.0 (10.1–22.4), and aHR = 101.1 (31.7–324.3)). However, the risk of hospitalization for migraine or PD was similar between the groups, even after adjustments.

## 4. Discussion

In this nationwide study focusing on hospitalizations for neurological disorders and based on ICD-10 codes, dementia, MS, and peripheral neuropathy presented a higher risk of hospitalization in pSS patients. To our knowledge, our study is the largest one to screen hospitalizations for neurological disorders in a population of French pSS patients. The risk of hospitalization for dementia among pSS patients was higher in comparison with the control matched patients and remained significant after adjustments. This increased risk is similar to that observed in a previous Taiwanese study (*n* = 4756; aHR of 1.35 (1.18–1.54) [16], which was even higher among pSS Taiwanese patients without comorbidities (aHR 2.37 (1.83–3.03)) [17]. Due to the limitations associated with epidemiological studies on dementia, and to eliminate another classical cause of dementia, we excluded patients with PD. Considering the frequency of encephalitis and MS in pSS [18,19,20,21], and the potential weight of both diseases on cognitive functions, we included them as time-dependent covariates in our analysis, but the strength of the association did not change. This suggests that the association between dementia and pSS is not mediated by encephalitis or MS.

Since mild cognitive impairment often precedes dementia [22], pSS patients might exhibit a higher prevalence of cognitive symptoms and impairments before hospitalization for dementia. Indeed, Massara et al. identified six patients with cognitive impairments (not responding to dementia criteria) among their 424 pSS patients, which was severe in one case [23]; and Jamilloux et al. found two patients among their 420 pSS patients with severe cognitive dysfunctions [24]. Notably, these early cognitive troubles are detectable only in clinical series with extensive neuropsychological investigations. Importantly, they would not be a cause of hospitalization (and, thus, are not captured in our results) but have the potential to strongly increase health consumption, hospitalization rates, and patient complaints (e.g., “brain fog”) [2]. Recent studies highlighted the urgent need to better characterize these cognitive impairments [25,26,27].

This may require an early multidisciplinary approach integrating neurologists and internists which focuses on these complaints and their course. An early screening of cognitive complaints through validated tools (such as the Brief Cognitive Symptoms Inventory (BCSI) or the Montreal cognitive assessment (MoCa)) could be used in daily practice [28,29]. In case of abnormalities, a complete neuropsychological work-up could help establish the impaired cognitive functions’ subdomains and how to treat them.

In addition to dementia, we confirmed the increased incidence of central nervous system (CNS) disorders in pSS patients. The leading causes of CNS-involvement in the disease appear to be myelitis, encephalitis, and abnormal white matter hyperintensities or “MS-like” lesions [18,19,20,21,23,24], all included in ESSDAI [19]. We found an increased hospitalization risk for encephalitis and MS. This is consistent with literature data [18,19,20,21], since the frequency of encephalitis has been reported from 1.4 to 2.4% [3,19,23,24] among pSS patients and leads to a wide spectrum of symptoms (e.g., headaches, cognitive impairments, and seizures).

The increased risk of MS in hospitalized pSS patients from our study raises several points. The mean age of diagnosis of MS in the general population (under 40 years old) is lower than in pSS patients (over 50 years) [30]. However, SS phenotypes have been reported among MS patients. Although a review reported a prevalence of SS between 0 and 3.3% among MS patients [31], some clinical series have reported a stronger association: de Sèze et al. observed 10 patients with suspected pSS among 60 patients with MS [32], and Annunziata et al. described 42 (9.5%) patients with clinical features of pSS [33] among their 440 MS patients (four of whom fulfilled the criteria for pSS). We thus assume that the higher hospitalization risk for MS in pSS patients directly translates into an association between the two diseases.

Moreover, several studies have reported abnormal white matter hyperintensities in pSS patients that sometimes resemble those seen in MS. Since most patients did not fulfill the MS criteria (including MRI criteria), they were considered as “MS-like” lesions [18]: among their 424 pSS patients, Massara et al. described five cases with “MS-like” CNS involvement of pSS [23], corresponding to 20% of all CNS involvement of the disease; Jamilloux et al. also described five patients with “MS-like” patterns among their 420 pSS patients [24]. Yet, in our epidemiological study based on ICD-10 codes, we cannot exclude that the CNS-involvement in SS might have been coded erroneously as “MS” in place of other conditions related to SS, especially white matter lesions of vascular origin, that could be frequent in pSS patients [34,35]. To attenuate the potential role of cardiovascular diseases and cardiovascular risk factors in MS or an “MS-like” diagnosis code, we also included cardiovascular diseases as adjusting factors in our models.

Overall, we suggest that MS could affect a subgroup of pSS patients. A bidirectional relationship may exist, as suggested by the Taiwanese study, in which first-degree relatives of pSS patients had a relative over-risk of 3.38 for MS [36]. Further prospective studies on large series of MS patients (involving immunological and radiological profiles of patients) could assess the strength of this association.

Both focal neurological and cognitive involvement of pSS raise the question of underlying pathophysiological pathways. Although MS and pSS are both treated with rituximab (anti-CD20 antibody, targeting B lymphocytes cells), no shared immunological dysregulated pathways have yet been identified. The potential role of encephalitis or MS in dementia onset was not demonstrated in our analysis. Because cardiovascular risk factors and diseases could also affect cognitive decline onset in pSS patients (vascular cognitive impairment), they were included as covariates in our analysis without changing the strength of the association. Other mechanisms, such as small vessel vasculitis targeting the CNS, could also be involved in dementia onset in pSS patients. Another underlying pathophysiological pathway linking cognitive decline and pSS could be interferon (INF) pathway dysregulations [37,38,39]. However, our preliminary data need further investigation to determine the exact underlying immunological mechanisms behind the dementia and cognitive troubles in pSS patients.

The prevalence rate of peripheral neuropathies in pSS is heterogeneous [24,40,41], but pure sensory neuropathy and axonal sensorimotor polyneuropathies are the two leading subtypes of this damage [42]. Here, we confirmed an increased risk of hospitalization for both inflammatory and non-inflammatory peripheral neuropathies among hospitalized pSS, but we could not associate specific neurological patterns (imprecise ICD-10 codes) with immunological and pathological pSS patterns (not available here).

Our study provides new insights about neurological conditions in pSS patients in France. One of its strengths is the number of individual observations selected from a nation-wide systematic health database, with the capacity to link time-scaled diagnoses at the individual level. This method allows associations of events with rare disease, such as pSS. However, we focused on in-patients in medicine departments. Yet, patients exhibiting mild cognitive impairment or moderate neurological disorders are often followed as outpatients and were not involved in our study. Thus, we could not study these early clinically-presenting forms of the diseases. However, this limitation also applies to the control group. Similarly, hospitalizations for psychiatric forms of dementia in psychiatric wards could not be studied. We also cannot exclude misdiagnosis for some patients, since our approach was based on ICD-10 codes, and treatment information was not available. Indeed, these codes are used for billing purposes and might not be strictly consistent with the diagnosis criteria of certain diseases such as MS, encephalitis, or peripheral neuropathies subtypes. Similarly, we also cannot certify that all pSS diagnoses fulfilled the ACR/EULAR criteria for pSS. Finally, we were not able to incorporate every confounding factor of interest in our incidence study, yet some confounders, such as alcohol consumption or educational level, could play a key role in dementia development. The cognitive consequences of past cerebrovascular diseases (such as stroke) could not be precisely identified in our study. Nonetheless, we have integrated past hospitalizations for cardiovascular risks and diseases, psychiatric disorders, and LSS in our models, which are known to be associated with dementia risk and could be involved in “MS-like” pathophysiology. As in most observational studies, we cannot exclude the possibility of residual confounding bias in our results.

## 5. Conclusions

Our nationwide study showed that hospitalization for dementia is at increased risk in pSS patients. This could partially be associated with the frequency of early cognitive complaints in pSS patients, such as “brain fog”. Further studies are needed to better characterize these cognitive symptoms and their underlying mechanisms, especially concerning the potential role of interferons. We also showed that pSS patients exhibit a higher incidence of hospitalization (according to ICD-10 codes) for peripheral neuropathies (as expected), encephalitis, and MS, which merit further investigations. Physicians must be aware of these over-risks to conduct an extensive diagnostic approach and characterize the neurological involvement of pSS.

## Figures and Tables

**Table 1:** Demographic and social characteristics of hospitalized primary Sjögren’s syndrome patients and their matched controls and adjustment factors.

	Sjögren’s Syndrome Patients (*n* = 25,661)		Matched Patients (*n* = 252,543)		
	Number of Patients	% of pSS Patients	Number of Matched Patients	% of Matched Patients	Comparison (*p* Value)
Sex (female) (*n*, %)	22,489	87.66%	224,887	87.65%	0.995
Low socio-economic status (*n*, %)	1224	4.77%	8482	3.31%	<0.001
Mean age (mean, SD)	60.2	±16.3	60.0	±16.3	0.075
Age ranks, years (*n*, %)					1
00–17	106	0.41%	1070	0.42%	
18–29	881	3.43%	8812	3.43%	
30–39	2123	8.27%	21,219	8.27%	
40–49	3374	13.15%	33,737	13.15%	
50–59	5287	20.61%	52,874	20.61%	
60–69	5933	23.13%	59,338	23.13%	
70–79	4697	18.31%	46,965	18.31%	
80–89	2878	11.22%	28,774	11.22%	
>90	377	1.47%	3771	1.47%	
Mean number of hospitalizations before index date (mean, SD)	3.7	± 9.0	0.21	±1.1	<0.001
Annual rate of hospitalizations before index date (*n*, %)					
≤0.25 per year	10,245	39.93%	244,107	95.15%	<0.001
between 0.25 and 0.5 per year	5959	23.23%	8679	3.38%	
between 0.5 and 1 per year	5430	21.16%	2807	1.09%	
between 1 and 5 per year	3704	14.44%	908	0.35%	
More than 5 per year	318	1.24%	59	0.02%	
Deaths (incidence ^#^, CI)	14.4	(13.7–15.2)	10.5	(10.3–10.7)	<0.001
Follow-up time after index date (median, (IQR), years)	3.96	(1.96–5.96)	3.96	(1.96–6.04)	0.003 *
Diseases’ codes reported in hospitalization stays prior to incidence study period (used as adjustment covariates)					
High blood pressure	274	1.07%	884	0.34%	<0.001
Diabetes	657	2.56%	2852	1.11%	<0.001
Obesity	294	1.15%	1395	0.54%	<0.001
Cardiovascular diseases	1360	5.30%	8828	3.44%	<0.001
Parkinson’s disease	56	0.22%	154	0.06%	<0.001
Multiple sclerosis	194	0.76%	264	0.10%	<0.001
Encephalitis	107	0.42%	48	0.02%	<0.001

pSS, primary Sjögren’s syndrome; *n*, number; SD, standard deviation; ^#^ number of incident deaths per 1000 person-years; all comparisons were performed with a Chi2 test, except those with a star (*), which were performed with Wilcoxon-Mann-Whitney rank-sum test.

**Table 2:** Hospitalization risks for neurological diseases in primary Sjögren’s syndrome patients compared to hospitalized matched patients.

	pSS Patients				Matched Patients									
	Incident Cases ^#^	Py	Incidence	CI	Incident Cases ^#^	Py	Incidence	CI	Crude HR	Crude CI	Crude *p* Value	Adjusted HR	Adjusted CI	Adjusted *p* Value
Dementia	207	101,420	2.04	(1.76–2.32)	1466	1,006,672	1.46	(1.39–1.53)	1.37	(1.18–1.58)	0.000	1.27	(1.04–1.55)	0.018
Multiple sclerosis	58	101,894	0.57	(0.42–0.72)	128	1,014,654	0.13	(0.11–0.15)	4.58	(3.35–6.25)	0.000	3.66	(2.35–5.68)	<0.001
Encephalitis	25	102,410	0.24	(0.15–0.33)	54	1,019,787	0.05	(0.04–0.06)	4.68	(2.9–7.55)	0.000	2.66	(1.22–5.80)	0.014
Parkinson’s disease	28	102,600	0.27	(0.17–0.37)	209	1,021,082	0.2	(0.17–0.23)	1.39	(0.93–2.06)	0.107	1.16	(0.66–2.04)	0.618
Migraine	36	102,456	0.35	(0.24–0.46)	174	1,019,354	0.17	(0.14–0.20)	2.02	(1.41–2.91)	0.000	1.44	(0.87–2.37)	0.157
Inflammatory polyneuropathies	67	101,892	0.66	(0.5–0.82)	36	1,016,623	0.04	(0.03–0.05)	18.04	(12.0–27.1]	0.000	23.22	(12.2–44.5)	<0.001
Other polyneuropathies	164	100,903	1.63	(1.38–1.88)	69	1,009,270	0.07	(0.05–0.09)	23.53	(17.7–31.4)	0.000	15.03	(10.1–22.4)	<0.001
Polyneuropathies in other classified diseases	63	102,095	0.62	(0.47–0.77)	5	1,018,421	0.00	(0.00–0.00)	150.39	(54.8–413.8)	0.000	101.1	(31.7–324.3)	<0.001
Trigeminal neuralgia	14	102,735	0.14	(0.07–0.21)	35	1,022,893	0.03	(0.02–0.04)	4.24	(2.26–7.95)	0.000	2.46	(0.9–6.7)	0.078

pSS, primary Sjögren’s syndrome; CI; confidence interval; py, person-years; ^#^ number of incident cases per 1000 person-years; HR, hazard ratio; aHR, adjusted hazard ratio. Adjusting factors combined socioeconomic status, annual rate of hospitalizations before index date, reported past diagnoses of diabetes, hypertension, obesity, and cardiovascular diseases. For dementia study, patients with Parkinson’s disease were excluded.

## Data Availability

Anonymized data access and data management programs are available on request to the corresponding author.

## Supplementary Materials

The following are available online at https://www.mdpi.com/article/10.3390/jcm11071979/s1. Table S1: Autoimmune conditions classifying Sjögren’s syndrome as secondary, and thus leading to the exclusion of the patient from the studied population, Table S2: Codes of the studied conditions, according to international classification of diseases (ICD-10), as searched in the national health database.
