# Supplementary material for: Hospitalization Risks for Neurological Disorders in Primary Sjögren’s Syndrome Patients
Source: J Clin Med. 2022 Apr 1;11(7):1979. doi: 10.3390/jcm11071979 (PMC9000167; doi:10.3390/jcm11071979)
Supplement: Supplementary file 1 [file jcm-11-01979-s001.zip › jcm-1626926-supplementary.pdf]

Supplementary Materials

Supplementary Table S1. Autoimmune conditions classifying Sjögren's syndrome as secondary, and thus leading to the exclusion of the patient from the studied population.

**ICD-10 code**	**Corresponding disease**
M069	Rheumatoid arthritis
M068	Other rheumatoid arthritis
M063	Rheumatoid nodule
M060	Seronegative rheumatoid arthritis
M080	Juvenile rheumatoid arthritis or juvenile arthritis
M05	Seropositive rheumatoid arthritis
L930	Systemic lupus erythematosus
L931	Cutaneous subacute lupus erythematosus
M321	Systemic lupus erythematosus with organ involvement
M328	Other systemic lupus erythematosus
M348	Other systemic sclerosis
M349	Systemic sclerosis
M332	Polymyositis
M308	Other disease mimicking polyarteritis nodosa
M316	Giant cell arteritis
M314	Takayasu's large-vessel vasculitis
M303	Kawazaki's syndrome
M300	Polyarteritis nodosa
L959	Skin vasculitis
M313	Granulomatosis with polyangiitis
M317	Microscopic polyangiitis
M301	Eosinophilic Granulomatosis with Polyangiitis
D690	Allergic purpura
M318	Other necrotizing vasculitis
M352	Behçet's disease
M090	Juvenile polyarthritis with psoriasis
M023	Reactive arthritis / Reiter's syndrome
B171	Hepatitis C
D86	Sarcoidosis
K50	Crohn's disease
K51	Ulcerative colitis
B20	HIV infection with infectious disease
B24	HIV infection
E85	Amyloidosis

ICD, international Classification of diseases

Supplementary TableS2. codes of the studied conditions, according to international classification of diseases (ICD-10), as searched in the national health database.

**Neurological conditions**	
Dementia	F00, F01, F02, F03
Multiple sclerosis	G35
Encephalitis or myelitis	G04
Parkinson's disease	G20
Migraine	G43
Inflammatory polyneuropathies	G618, G619
Other polyneuropathies	G628, G629
Polyneuropathies in other classified diseases	G635, G638
Trigeminal neuralgia	G500
**Covariates**	
Hypertension	I10, I15
Diabetes	E10, E11, E12, E13, E14
Obesity	E66
Cardiovascular diseases (ischemic heart disease, stroke, aortic dissection, aortic and peripheral arterial disease, and hypertensive chronic kidney disease)	I20, I21, I22, I23, I24, I25, I70, I71, I63, I64
Psychiatric disorders (depression, anxiety)	F32, F33, F40, F41
